# Error in the Byline, Abstract, and Results

**DOI:** 10.1001/jamanetworkopen.2025.0680

**Published:** 2025-02-07

In the Special Communication titled, “Digital Health Technology Research Funded by the National Institutes of Health,”^1^ published on January 3, 2025, Dr Doyle’s first name was misspelled. The name should appear as Jamie Mihoko Doyle, PhD. In the Abstract, the percentage of the digital health technology portfolio (DHT) was incorrect. The DHT portfolio represented 2.8% (US $7 628 967 500) of the overall National Institutes of Health (NIH) grants funded from 2015 to 2023. In the Results section, the NIH’s investment in DHT and the budget for 2015 to 2023 were incorrect. This amount was 2.8% of the total NIH budget of $272 910 880 000 for this period. In addition, the percentage of the activity codes for T32s should be 5.7%. This article has been corrected.

## References

[1] Cure P, Radman T, Doyle JM, Atienza AA, Fessel JP, Hartshorn CM. Digital health technology research funded by the national institutes of health. JAMA Netw Open. 2025;8(1):e2452976. doi:10.1001/jamanetworkopen.2024.52976 39752153

